# Death from Chloroform

**Published:** 1860-10

**Authors:** 


### ARTICLE XV.

### *Death from Chloroform.*

ANOTHER death from chloroform is reported in the *Cincinnati Lancet and Observer*, by Dr. KRAUSE, of Cincinnati. It occurred during an operation for iridectomy. The following is an abstract of the case in Dr. K's own words:

"On the 25th of last month I performed an operation for artificial pupil, on a farmer, 29 years of age, who had generally enjoyed good health. About a year ago he suffered from protracted intermittent fever. The disease of his eyes dated from this time. His constitution was scrofulous anæmic. Preparatory to the operation chloroform was administered on a folded cloth, by an assistant sufficiently expert in its use. The patient, who had been enjoined to keep his stomach empty on the morning of the operation, inhaled the chloroform in the recumbent posture, from 11 to about 11½ o'clock. He took one and a half ounces of it without resistance; nor did he even manifest the usual ecstatic symptoms. I finally proceeded with the operation, after having three or four times desisted from it on account of the patient's restlessness whenever the lid-holders were applied. Previously, however, the removal of the chloroform was ordered from the patient's mouth and nose, as his breathing had begun to be stertorous. The operation, iridectomy, lasted about five minutes. The anterior chamber of the eye partly filled with blood, which I was about to let out, when I noticed a sudden paleness of the anterior ciliary vessels, which had become injected under the touch of the instruments. Then I found that the patient had ceased to respire, while the action of his heart, though weak, was still perceptible to the ear, regular, and about sixty beats in a minute. Ice-water, sprinkled into the patient's face, on his chest and epigastrium, had no effect. Rhythmical depression of the abdomen also failed to restore respiration. I therefore resorted to Marshall Hall's method. Windows and doors were opened, the patient's mouth and throat cleaned from a very tenacious mucus, which was not prone to discharge by its own gravity, and peripherical circulation promoted by rubbing of the extremities heartward, the occasional use of ice-water and clapping of the skin. The function of the heart was sustained by these means nearly an hour. Respiration, however, which had occurred at first about once every minute a few times, gradually lessening the patient's livid complexion, became less frequent and more superficial, until it degenerated into mere pseudo-pnoic efforts. There was during the agony a twitching of the muscles about the mouth, and a drawing up of the patient's legs: then pulsation also stopped. We ceased our efforts at resuscitation one hour and a quarter after the first symptoms of apnœa had appeared.

"No post mortem examination was made. The deceased had never complained of anything indicating disease of his thoracic organs. His size was over six feet, the configuration of his chest normal. During my short acquaintance with him his spirits were depressed, his temper torpid."

There is no comment necessary. So rapidly are the cases accumulating, showing the often unavoidable fatality of chloroform, that hereafter the administration of chloroform, except in cases where ether has absolutely failed, must be considered as a sign of recklessness or ignorance.

*Med. and Surgical Reporter.*

